# Three-dimensional protein model similarity analysis based on salient shape index

**DOI:** 10.1186/s12859-016-0983-z

**Published:** 2016-03-18

**Authors:** Bo Yao, Zhong Li, Meng Ding, Minhong Chen

**Affiliations:** Departments of Mathematical Sciences, Zhejiang Sci-Tech University, Hangzhou, 310018 China

**Keywords:** Protein model, Shape index, Salient geometric feature, Shape analysis

## Abstract

**Background:**

Proteins play a special role in bioinformatics. The surface shape of a protein, which is an important characteristic of the protein, defines a geometric and biochemical domain where the protein interacts with other proteins. The similarity analysis among protein models has become an important topic of protein analysis, by which it can reveal the structure and the function of proteins.

**Results:**

In this paper, a new protein similarity analysis method based on three-dimensional protein models is proposed. It constructs a feature matrix descriptor for each protein model combined by calculating the shape index (SI) and the related salient geometric feature (SGF), and then analyzes the protein model similarity by using this feature matrix and the extended grey relation analysis.

**Conclusions:**

We compare our method to the Multi-resolution Reeb Graph (MRG) skeleton method, the L1-medial skeleton method and the local-diameter descriptor method. Experimental results show that our protein similarity analysis method is accurate and reliable while keeping the high computational efficiency.

## Background

Protein similarity analysis is an important topic in bioinformatics. With it, we can help understand the structure and the function of proteins. The protein shape analysis plays an important role in medical research, computer aided molecular design, protein structure retrieval and prediction, among others. However, the analysis is highly challenging due to the complexity of a protein’s three-dimensional surface shape, which can deform significantly enough to change the topological structure during molecular interactions [1].

Many researchers have contributed to similarity analysis methods for comparing protein shapes. Via et al. [2] gave a survey on the current knowledge of the protein surface similarity. The similarity analysis method based on comparison of shape feature is a common approach. Sael et al. [3] proposed a popular protein surface similarity method based on a 3D Zernike descriptor. Compactness and rotational invariance of this descriptor enable fast comparison suitable for protein database searches. However, in order to capture the high resolution of the protein surface similarity, computation increases as the number of terms in its series expansion increases. And it is not applicable for the protein models with complex topology such as holes. Osada et al. [4] provided a shape distribution method based on the statistical histogram that measures the vertex distribution of the whole model surface, from which it forms a shape feature distribution histogram, and finally obtains a three-dimensional model’s geometric similarity measure by comparing two similar distances. Horn et al. [5] proposed an algorithm based on an extended Gaussian image, in which it maps each grid of the model surface to a unit sphere, thus obtains an extended Gaussian ball vector. Ohbuchi et al. [6] presented a statistical histogram algorithm in which the three-dimensional model vertices are sampled and then a three-dimensional coordinate axis histogram is used to generate three statistics about the model’s geometric features. Vranic et al. [7] introduced a functional analysis method that assesses the three-dimensional model similarity using the modulus of a spherical harmonic analysis coefficient.

Other shape similarity methods based on topology are also widely studied. For example, Hilaga et al. [8] proposed a multi-resolution Reeb graph (MRG) method. It uses the model surface’s geodesic distance as a Morse function to draw the multi-resolution Reeb graph of a three-dimensional model. Bronstein et al. [9] provided a method based on heat kernel signatures (HKS). It draws analogies with feature-based image representations to construct shape descriptors, which are invariant to a wide class of transformations on one hand and are discriminative on the other hand. Forked et al. [10] proposed a method based on the simplified medial axis, which is parameterized by a separation angle. The angle is formed by the vectors connecting a point on the medial axis to the closest points on the boundary. Du et al. [11] proposed a method based on the skeleton graph. It first calculates the skeleton node of a three-dimensional model and then constructs the corresponding skeleton graph between nodes. Both of these methods are computationally expensive, are more sensitive to holes in the three-dimensional models, and are lack of robustness to noise. Morris et al. [12] obtained a similarity comparison of three-dimensional protein models by spherical harmonic expansion. Fang et al. [13] proposed a shape comparison method based on the local diameter (LD). Qin et al. [14] introduced an improved MRG skeleton algorithm. In this process, sample points are used to build the local diameter (LD) for a model similarity comparison, but it is a computationally expensive approach. Li et al. [15] presented a method based on an improved L1-medial protein skeleton, but they only apply it to the CPK protein model. Hence, their method is not generally applicable to all three-dimensional protein models.

Motivated by the salient theory of a three-dimensional model proposed by Hoffman and Singh [16], and by the shape index (SI) concept by Bradford et al. [17], we propose a new shape comparison method for three-dimensional protein models. We first compute the shape index which reflects the protein surface’s geometric feature including the concave and convex properties. Then we construct the salient geometric feature (SGF) through the region-related shape index information. The shape index and the salient geometric feature are then combined to form the feature matrix of each protein model. We finally use the extended grey relation analysis to analyze the feature matrix and obtain the final shape similarity results of protein models.

## Methods

**Fig. 1:**
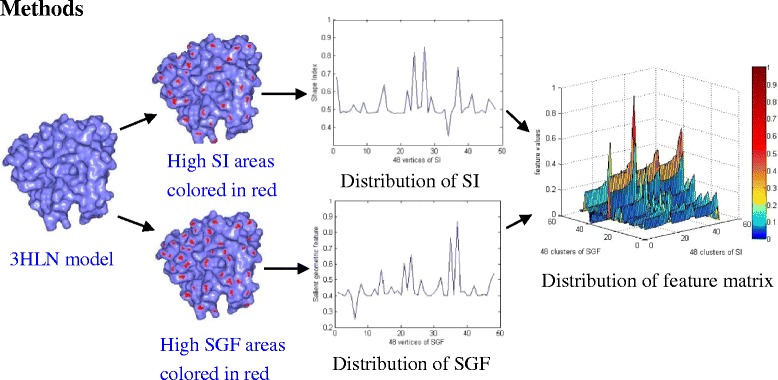
Main steps of our similarity analysis for protein models. For a given 3HLN model, the high SI and SGF areas are colored in red on the 3HLN surface, respectively. The distribution of feature matrix is constructed by the SI and SGF distribution vectors

A three-dimensional protein model can be represented by the form with the triangular mesh. We first estimate the curvature of each vertex on a protein model surface, and calculate the shape index (SI) and the salient geometric feature (SGF) based on the shape index of each vertex. Then, we construct the protein model’s feature matrix through the shape index and the salient shape index. Finally, we do the similarity analysis for protein models by the matrix-based grey relation analysis. The main process of our algorithm is shown in Fig. 1.

### Shape index (SI)

The concept of shape index was proposed in [17]. It is a curvature-related parameter that describes the protein surface’s concave and convex properties. As we know, surface curvature controls the surface orientation and provides information about its degree of concavity or convexity. Thus, the shape index is thought to play an important role in determining the stability of the protein molecules in the process of molecular recognition and structure prediction. The shape index of a protein model can help us study the atomic-level geometry of the interacting versus non-interacting regions of a protein, and therefore help us understand protein interaction mechanisms. Here, we focus on using the shape index to represent the shape characteristics of a protein surface. The shape index (SI) of a protein model is defined as

$$SI=-\frac{2}{\pi } \arctan \frac{k_1+{k}_2}{k_1-{k}_2},$$

where *k*_1_ and *k*_2_ denote the maximum and the minimum principal curvatures, respectively. From the above formula, we know SI is between -1 and 1. When the shape index is close to 1, it indicates the convex shape of the given vertex on the protein surface. On the contrary, when the shape index is close to -1, it indicates the concave shape of the given vertex on the surface. When *k*_1_ = - *k*_2_, the shape index is 0.

SI relates to the curvature estimation of each vertex on the model surface. We use Dyn and Hormann’s method [18] to estimate the discrete Gaussian curvature *k*_*G*_ and the discrete mean curvature *k*_*M*_. Then, the maximum principal curvature *k*_1_ and the minimum principal curvature *k*_2_ are obtained by

$${k}_1={k}_M+\sqrt{{k_M}^2-{k}_G},\kern0.5em {k}_2={k}_M-\sqrt{{k_M}^2-{k}_G}.$$

**Fig. 2:**
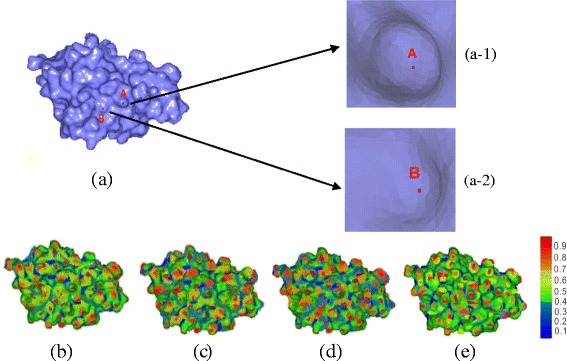
1HLB protein model and its geometric feature models. **a** 1HLB protein model. (a-1) Local area of the vertex *A*. (a-2) Local area of the vertex *B*. **b** Gaussian curvature model of 1HLB protein. **c** Mean curvature model of 1HLB protein. **d** Shape index model of 1HLB protein. **e** Salient geometrical feature model of 1HLB protein

For the 1HLB protein model in Fig. 2a, we give the corresponding Gaussian curvature figure and mean curvature figure as shown in Fig. 2b and c, where the red and the blue areas represent large and small curvature regions, respectively. We also show the corresponding shape index figure in Fig. 2d, where the red and the blue areas represent convex and concave regions, respectively.

### Salient Geometric Feature (SGF)

Salient geometric feature is built on the theory of salience of visual parts proposed by Hoffman and Singh [16]. They regarded that the salience of a part depends on two factors: its size relative to the whole object, and the number of curvature changes and their strength. This concept has been applied to three-dimensional mesh model matching [19]. It constructed a salient feature formula based on geometric information, which can detect some areas that the topology and numerical calculations may not be similar, but they are considered to be substantially similar. Here, we focus on using the shape index to construct the salient shape index of a protein surface. It similarly includes the local area size and its shape index variance by

$$SGF={\displaystyle \sum_{i\in F}{w}_1 Area(i)SI{(i)}^3+{w}_2N(SI)Var(SI)},$$

where *F* is a cluster consisting of each vertex *i*, *w*_1_ and *w*_2_ are the weights, we set them as 0.5. *Area*(*i*) is the area of the patch associated with vertex *i* relative to a cluster size, *N*(*SI*) is the number of local minimum(s) or maximum(s) shape index in the cluster, *Var*(*SI*) is the shape index variance in the cluster, *SI*(*i*) is the shape index associated with vertex *i*.

For the 1HLB protein, we give its salient geometric feature model in Fig. 2e. The regions with the red color represent the more salient parts, and the regions with the blue color are the less salient parts. And we also use the 1HLB model as the example to address the difference scales of SI and SGF on the protein surface. For vertex A in Fig. 2(a-1), its SI value is 0.5368 and its SGF value is 0.6425. For vertex B in Fig. 2(a-2), its SI value is 0.5279 and its SGF value is 0.2981. We find their SI values are close which are hard to reflect the difference of local feature. Whereas, their SGF values have a big difference because SGF value is related to the local geometric region. When the local geometric region varies saliently, the SGF value is high. So from this model, we conclude that point A has a salient geometric feature since it has a high SGF value.

### Feature descriptor structure

The shape index and the salient geometric feature of all vertices on a three-dimensional model constitute an *n*-dimensional vector (where *n* is the number of model vertices), respectively. Because the number of vertices on each model surface is not the same for different proteins, these vectors cannot be directly compared and analyzed. In our approach, we cluster all feature values into the same group number through a clustering algorithm [20]. For the number of clusters representing different features in *K*-means clustering, the high value of *K* will improve the accuracy of shape analysis, but it also increases the computation of shape comparison. The low value of *K* does not need the high running time of computation, but it can not guarantee the accuracy of shape analysis. Here, we set it as *K* = 48. Then, we calculate the mean for each group *t*_*i*_ and obtain a feature described vector of shape indexes *T* = (*t*_1_, *t*_2_, …, *t*_*K*_). For the shape index feature clustering of a protein model, we randomly select *K* data points from the database of *n* values as the initial cluster centers for use with the *K*-means clustering algorithm. We perform clustering until the change in cluster centers reaches a convergence condition. From this, we obtain the final *K* data point clusters.

**Fig. 3:**
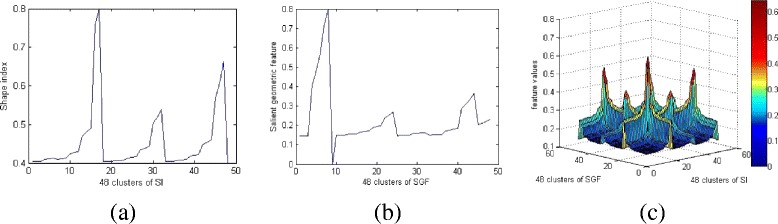
Feature distribution figures of 1HLB protein model. **a** Shape index feature distribution. **b** Salient geometric feature distribution. **c** Feature distribution combination model of 1HLB protein model

Similarly, the salient geometric features of a protein model can be represented as a vector *P* = (*p*_1_, *p*_2_, …, *p*_*K*_). The shape index feature and salient geometrical feature of the 1HLB protein model are shown in Fig. 3a and b, where the horizontal axis represents 48 representative groups obtained by clustering, and the ordinate axis represents the features. We notice that there is no correspondence between clusters in Fig. 3a and b, because each cluster is determined by the randomly selected initial vertices on the protein surface.

In order to better reflect the shape feature of a three-dimensional protein model, the method based on the feature matrix expression has become a popular method for the model shape analysis [21]. Here, we apply above two vectors to construct a matrix which contains rich feature information as a feature descriptor. We denote *Q*_*K*×2_ = [*T*; *P*]^*T*^ and use *Q*^*T*^*Q* to represent a *K* × *K* (*K* = 48) feature matrix, then do the similarity analysis for the protein models with this feature descriptor. We give the feature matrix figure of 1HLB protein model in Fig. 3c.

### Similarity measurement

For the shape analysis of a protein sequence or its surface model, common methods use distance measurements such as Euclidean distance, Manhattan distance, angle cosine method and correlation coefficient method, etc [22]. One problem using these methods is that the measure value is normally not guaranteed to lie in the standard interval [0, 1]. If we use the normalization to transform the values into [0,1], it relates to the maximum and the minimum measure values of all protein models and this transformation will influence the accuracy of the shape analysis for protein models.

For our similarity analysis of three-dimensional protein models, because we construct a matrix-based feature descriptor, the previous vector-based method is not directly applicable for our measurement. At the same time, we hope to advocate the use of a scalar value of similarity directly between 0 and 1, where higher values represent greater similarity between two protein models. Here we popularize the vector-based grey relation analysis [23] to the matrix-based grey relation analysis, which also keeps the value in [0,1] and other properties of the grey relation analysis. Then, we apply it to measure the similarity of three-dimensional protein models.

Suppose that *X* and *Y* are matrices with the same *m* rows and *n* columns

$$X={\left[x\left(i,j\right)\right]}_{m\times n},\kern0.5em Y={\left[y\left(i,j\right)\right]}_{m\times n},\kern0.5em i\kern0.5em =\kern0.5em 1,2,\kern0.5em \cdot \cdot \cdot \kern0.5em ,\kern0.5em m,\kern0.5em j\kern0.5em =\kern0.5em 1,2,\kern0.5em \cdot \cdot \cdot \kern0.5em ,n.$$

For the *k*^th^ row, we produce the image of zero starting point of matrix *X* and *Y*

$${x}^{\hbox{'}}\left(k,q\right)=x\left(k,q\right)-x\left(k,1\right),\kern0.5em {y}^{\hbox{'}}\left(k,q\right)=y\left(k,q\right)-y\left(k,1\right),\kern0.5em q\kern0.5em =\kern0.5em 1,2,\kern0.5em \cdot \cdot \cdot \kern0.5em ,n.$$

Then, we compute the grey relation degree of matrix *X* and *Y* for each row

where $${\varepsilon}_{ij}^m(k)=\frac{1+\left|s(k)\right|+\left|t(k)\right|}{1+\left|s(k)\right|+\left|t(k)\right|+\left|s(k)-t(k)\right|},$$

$$s(k)={\displaystyle \sum_{q=1}^n{x}^{\hbox{'}}\left(k,q\right)},\kern0.5em t(k)={\displaystyle \sum_{q=1}^n{y}^{\hbox{'}}\left(k,q\right)},\kern0.5em k\kern0.5em =\kern0.5em 1,2,\kern0.5em \cdot \cdot \cdot \kern0.5em ,n.$$

Similarly, we get the grey relation degree *ε*_*ij*_^*n*^(*k*) of matrix *X* and *Y* for the *k*^th^ column. Finally, we obtain the grey relation degree of matrix *X* and *Y* by

$$\eta =\frac{1}{2}\left(\frac{1}{m}{\displaystyle \sum_{k=1}^m{\varepsilon}_{ij}^m(k)}+\frac{1}{n}{\displaystyle \sum_{k=1}^n{\varepsilon}_{ij}^n(k)}\right).$$

From the above calculation process, it is easily known that the grey relation degree is between 0 and 1, and the degree indicates the high similarity of two models when it is close to 1.

## Results

The algorithm presented in this paper is implemented on a Intel(R) Core(TM) i3-3110 M CPU @2.5 Ghz desktop computer with 4GB RAM running MS Windows 7. The software environment of the experiment is based on Mathworks’ MATLAB R2010a.

**Fig. 4:**
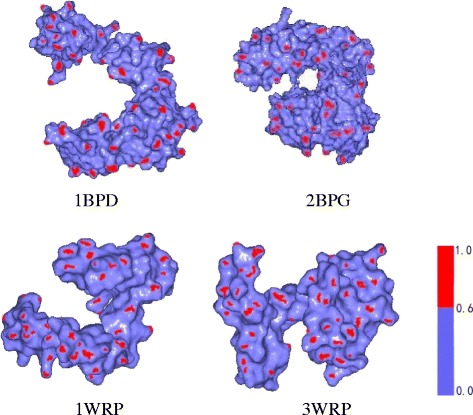
Four protein models (1BPD, 2BPG, 1WRP and 3WRP). Red regions represent salient shape index areas

We first chose four protein models from the Protein Data Bank [24], which are shown in Fig. 4. We already know that the 1BPD and 2BPG models are similar and the 1WRP and 3WRP models are similar [15]. Table 1 shows the results of comparing our algorithm with Qin et al’s algorithm [15] which is based on the improved L1-medial skeleton extraction. The similarity measurement values of two methods are both between 0 and 1, and the more similar two protein models, the more close to 1 their values. Our analysis method obtains a reasonable similarity comparison result because our value is closer to 1 by comparing bold data in Table 1. In Table 2, we compared the execution time of two algorithm’s implementations, which shows that our method runs faster than Qin et al’s algorithm [15]. We also compare our matrix-based feature descriptor to the vector-based method directly by SI, SGF, and the simply combined feature vector method ((SI + SGF)/2). The results are shown in Table 3. For two pairs of similar proteins, we find our similarity result is more close to 1.

**Table 1:** Comparison by the grey relation distance between Qin et al’s algorithm [15] and our algorithm

	Similarity values by different algorithms		
1BPD	2BPG	1WRP	3WRP
Algorithm in [15]	**0.9713**	0.5625	0.5816
Our algorithm	**0.9856**	0.3853	0.3605
2BPG	1BPD	1WRP	3WRP
Algorithm in [15]	**0.9713**	0.6497	0.6823
Our algorithm	**0.9856**	0.3844	0.3596
1WRP	1BPD	2BPG	3WRP
Algorithm in [15]	0.5625	0.6497	**0.9597**
Our algorithm	0.3853	0.3844	**0.9723**
3WRP	1BPD	2BPG	1WRP
Algorithm in [15]	0.5816	0.6823	**0.9597**
Our algorithm	0.3605	0.3596	**0.9723**

Bold numbers mean the similarity values measured by different methods for similar proteins

**Table 2:** Running time comparison between Qin et al’s [15] algorithm and our algorithm (The time unit is ms)

Model	1BPD and 2BPG		1WRP and 3WRP	
Time	Algorithm in [15]	Our algorithm	Algorithm in [15]	Our algorithm
	2153	112	5716	216

**Table 3:** Grey relation distance comparison by different similarity measure methods

Similarity measure methods	1BPD and 2BPG	1WRP and 3WRP
Feature vectors of SI (*T*)	0.9346	0.9517
Feature vectors of SGF (*P*)	0.9419	0.9486
Combined feature vectors of SI and SGF ((*T* + *P*)/2)	0.9357	0.9541
Matrix-based feature descriptor (*Q* ^*T*^ *Q*)	0.9856	0.9723

**Fig. 5:**
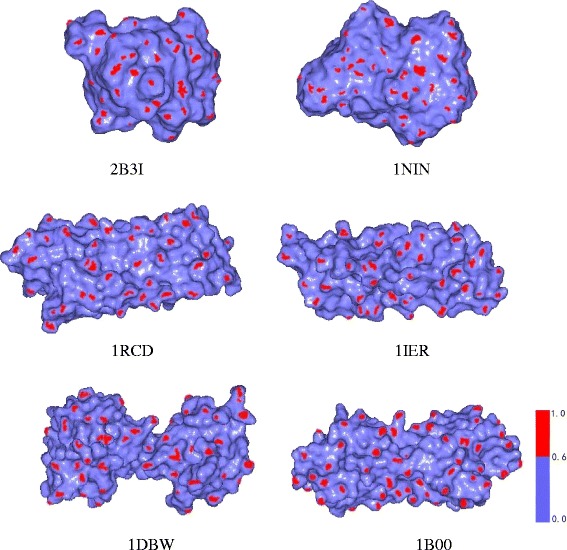
Six protein models from Skolnick dataset (2B3I, 1NIN, 1RCD, 1IER, 1DBW and 1B00)

Then, we chose three groups of protein models from the Skolnick dataset [25], which are shown in Fig. 5. We already know that the 2B3I and 1NIN proteins are similar because they are in the same clustering [14,25]. For other four protein models, the 1RCD and 1IER models are similar, and the 1DBW and 1B00 models are similar [14,15]. We used our method to compare similarities of these three pairs of proteins and find that they are in accordance with the results of [14,15,25]. Table 4 indicates that two models with bold underlined data are similar (The similarity of a model with itself is always 1.000). We also compared the execution time of Li et al’s method [14] based on the improved skeleton extraction by Reeb Graph and our method in Table 5. We find our method is obviously faster than the method in [14] because it does not need to conduct the skeleton extraction.

**Table 4:** Similarity results for six protein models from Fig. 5

Models	2B3I	1NIN	1RCD	1IER	1DBW	1B00
2B3I	1.0000	**0.9500**	0.8812	0.4460	0.9350	0.9244
1NIN	**0.9500**	1.0000	0.9269	0.4918	0.9241	0.9176
1RCD	0.8812	0.9269	1.0000	**0.9630**	0.9109	0.7513
1IER	0.4460	0.4918	**0.9630**	1.0000	0.4749	0.4785
1DBW	0.9350	0.9241	0.9109	0.4749	1.0000	**0.9930**
1B00	0.9244	0.9176	0.7513	0.4785	**0.9930**	1.0000

Bold numbers mean the similarity values measured by different methods for similar proteins

**Table 5:** Running time comparison between the skeleton extraction algorithm [14] and our algorithm

Model	2B3I		1NIN		1RCD		1IER		1DBW		1B00	
	A	B	A	B	A	B	A	B	A	B	A	B
2B3I	2480	104	2481	104	2465	105	2481	105	2480	104	2464	105
1NIN	7472	215	8472	202	7753	208	6131	210	11809	198	10733	205
1B00	7457	208	10733	205	7738	207	10743	200	10732	194	10749	202
1RCD	7488	210	7753	208	7737	212	7759	198	7753	207	7738	207
1DBW	7456	204	11809	198	7753	207	6130	194	11825	195	10732	194
1IER	7472	199	6131	210	7753	198	6147	194	6130	194	10732	200

Notes that A is the running time of algorithm in [14], B is the running time of our algorithm. The time unit is ms

**Fig. 6:**
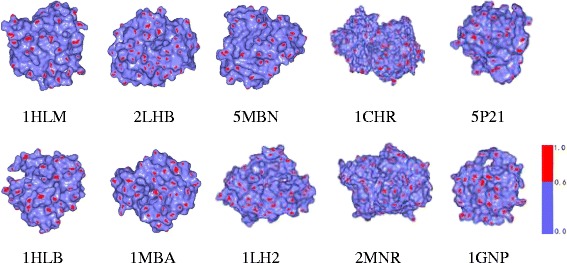
Ten protein models from Chew–Kedem dataset (1HLM, 1HLB, 2LHB, 1MBA, 5MBN, 1LH2, 1CHR, 2MNR, 5P21, 1GNP)

Next, we chose 10 protein models from the Chew–Kedem dataset [26], which are shown in Fig. 6. We have known that the 1HLM and 1HLB proteins are similar because they are in the globin family, the 5P21 and 1GNP proteins are similar because they are the alpha–beta family [26]. We computed the matrix-based grey relation distance by using our method. The similarity results of protein models corresponding to 10 proteins are shown in Table 6. We find our similarity results are in agreement with the results in [26].

**Table 6:** Similarity results for ten protein models from Fig. 6

Models	1HLM	1HLB	2LHB	1MBA	5MBN	1LH2	1CHR	2MNR	5P21	1GNP
1HLM	1.000	0.973	0.562	0.676	0.437	0.752	0.653	0.549	0.789	0.752
1HLB	0.973	1.000	0.608	0.763	0.574	0.647	0.564	0.653	0.742	0.698
2LHB	0.562	0.608	1.000	0.969	0.579	0.695	0.745	0.659	0.732	0.634
1MBA	0.676	0.763	0.969	1.000	0.614	0.713	0.697	0.705	0.720	0.744
5MBN	0.437	0.574	0.579	0.614	1.000	0.987	0.353	0.438	0.493	0.464
1LH2	0.752	0.647	0.695	0.713	0.987	1.000	0.434	0.468	0.464	0.413
1CHR	0.653	0.564	0.745	0.697	0.353	0.434	1.000	0.993	0.563	0.615
2MNR	0.549	0.653	0.659	0.705	0.438	0.468	0.993	1.000	0.595	0.685
5P21	0.789	0.742	0.732	0.720	0.493	0.464	0.563	0.595	1.000	0.981
1GNP	0.752	0.698	0.634	0.744	0.464	0.413	0.615	0.685	0.981	1.000

We also demonstrate a total running time including searching the most similar protein model for 5 protein models in the Chew-Kedem dataset. In Table 7, we find our method has a fast searching speed for obtaining the similar protein model.

**Table 7:** Running time comparison including searching the dataset between the skeleton extraction algorithm [14] and our algorithm

Models Methods	_1HLM_	_2LHB_	_5MBN_	_1CHR_	_5P21_
A	20mins 28 s	25mins 05 s	22mins 49 s	18mins 58 s	21mins 36 s
B	5mins	7mins	6mins	5mins	6mins
	43 s	19 s	57 s	34 s	26 s

Note that A is the algorithm in [14] and B is our algorithm

**Fig. 7:**
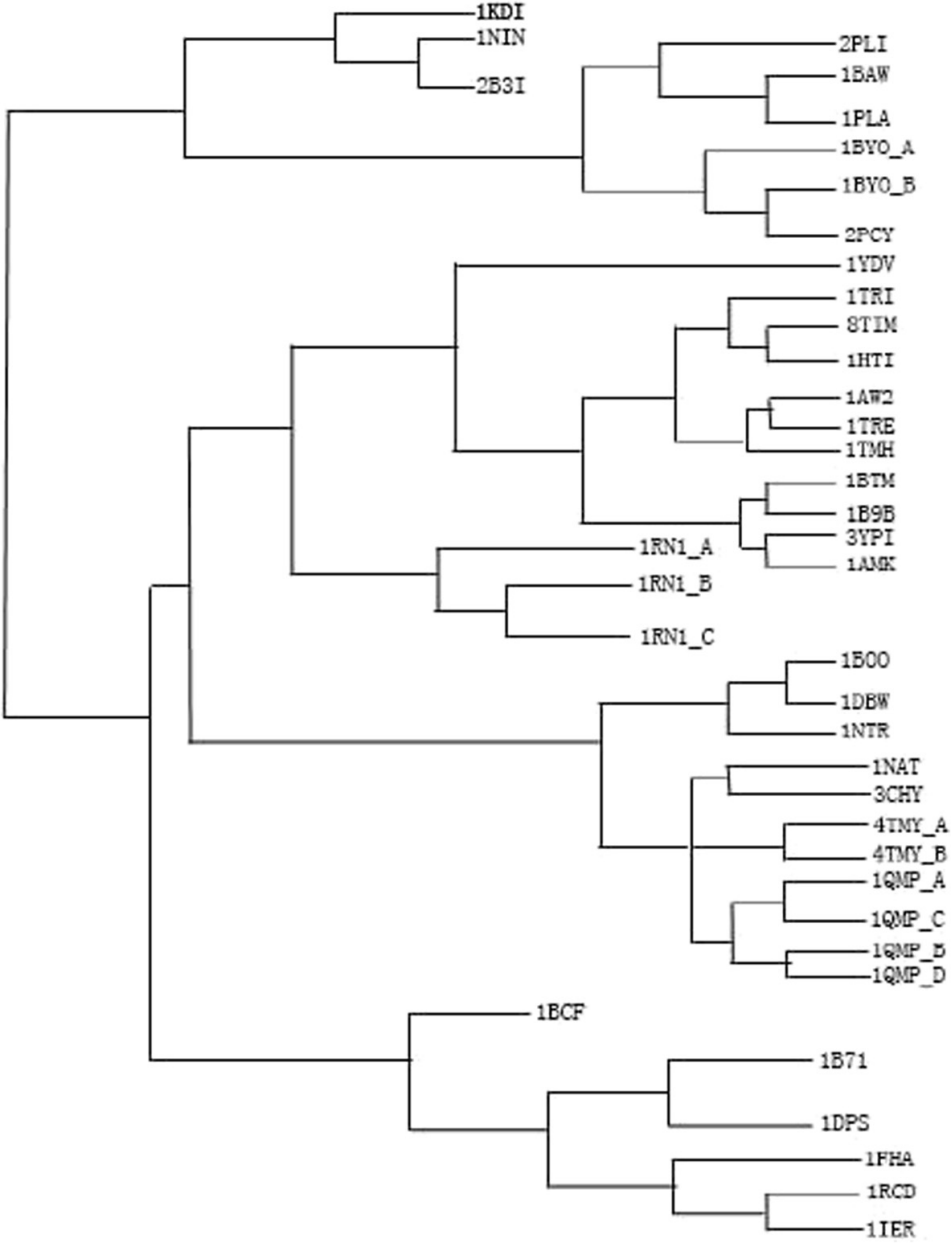
Average linkage of Skolnick's dataset by our method

To increase the robustness of our method, we added another testing dataset as Skolnick’s dataset from R[25] for the experiment, which includes 40 proteins models. We use our method to construct the average linkage of Skolnick's dataset in Fig. 7 and find that our result is almost consistent with the result in R[25]. For example, the 3YPI and 1AMK proteins are in the same cluster, the 1NAT and 3CHY proteins are in the same cluster. These results are in accord with the current evolutionary research [14,25].

**Fig. 8:**
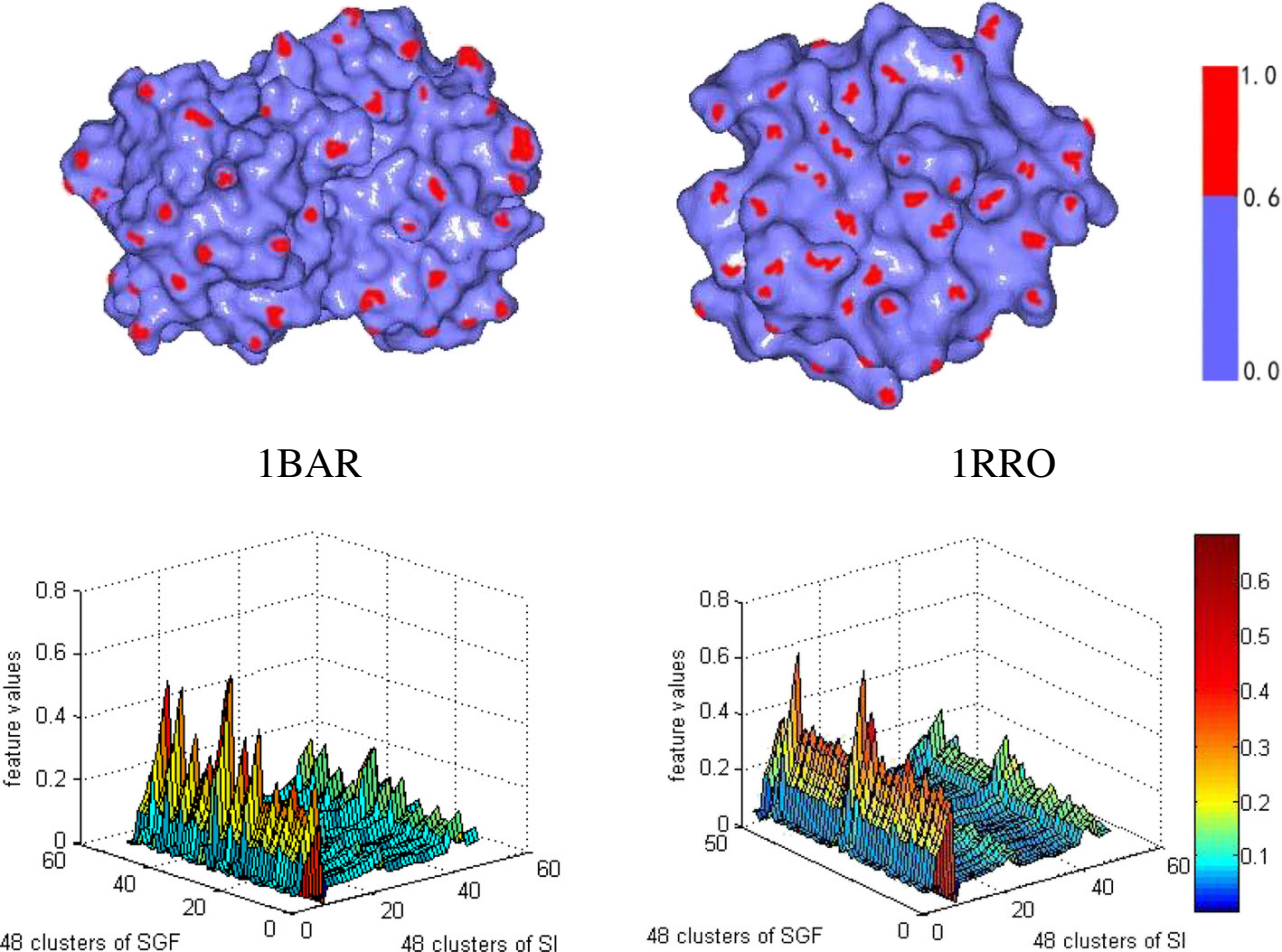
1BAR and 1RRO protein models with their feature distributions

Finally, we compared two proteins (1BAR and 1RRO) that have a similar shape surface but have completely different secondary structure elements [13] (Fig. 8). The algorithm [13] based on the shape analysis by the local diameter construction resulted in a similarity of 0.9956, which is close to 1. It falsely reflects the similarity properties of the two proteins with different secondary structures. Our method produced a similarity value of 0.7546 which is comparatively some smaller than 1. It can infer the non-similarity for two protein models although they have similar shape surface. We conclude that our approach, in this specific case, improves the similarity analysis for non-homologous protein models with similar shapes.

## Discussion

Our method is based on the surface analysis and the advantage is that the running time is fast because it does not need to conduct the skeleton extraction. The disadvantage is that it can not be applied for other protein models such as protein CPK models. The advantage of the skeleton based method is that it can be applied for both the protein surface (triangular mesh model) and the protein CPK model (point cloud representation), the disadvantage is the skeleton extraction requires a time-consuming process.

## Conclusion and future work

In this paper, we propose a three-dimensional protein model’s similarity analysis algorithm based on salient shape index. We first calculate the shape index (SI) and salient geometric feature (SGF) of the protein models. And then we construct the matrix-based feature descriptor by SI and SGF information. Finally, we compare the similarity of protein models by the matrix-based grey relation degree. Experimental results show the effectiveness of our protein similarity analysis method.

Currently, we only consider the shape index (convex and concave properties of the protein surface) and the salient geometric feature to analyze the similarity of the protein models. We do not take account of the physical properties of the protein molecules. In fact, these properties such as pH, polar and non-polar, hydrophilic, also affect the structure and the function of the protein molecules. How to combine these factors for the protein shape similarity analysis will be our future research. For the clustering in our similarity analysis, we find the cluster size is normally not equal and the clustering is sometimes dominated by several big clusters. The size of the clusters might be highly relevant in describing the global shape of the protein model. This also gives us an interesting work for our future research.
